# Case Report: Partial resection of lingual ectopic thyroid using an electrohook under laryngoscopic guidance

**DOI:** 10.3389/fsurg.2026.1783218

**Published:** 2026-05-13

**Authors:** Kepeng Li, Jinxiu Wang

**Affiliations:** 1 Department of Otorhinolaryngology, Jingmen Central Hospital, Jingmen, China; 2 Jingmen Central Hospital Affiliated to Jingchu University of Technology, Jingmen, China

**Keywords:** case report, cost-effectiveness, electrohook, hypothyroidism, lingual thyroid

## Abstract

**Introduction:**

Lingual thyroid is a rare ectopic thyroid disorder, with an incidence of 1 in 100,000–300,000. Surgical treatment options include transoral robotic surgery, transoral laser microsurgery, and coblation; however, these require expensive equipment, which may limit accessibility. We report a case that was successfully treated using an electrohook, a universally available and cost-effective alternative.

**Patient concerns:**

A 56-year-old woman presented with a 10-year history of foreign body sensation and dysphagia, which had recently worsened and was significantly affecting her quality of life, prompting her to seek medical attention.

**Diagnosis:**

Based on electronic laryngoscopy revealing a mass at the tongue base, elevated thyroid-stimulating hormone (TSH) levels (40 μIU/mL), a reduced cervical thyroid on ultrasonography, and contrast-enhanced CT demonstrating a characteristic thyroid-like enhancement pattern, a diagnosis of lingual ectopic thyroid with hypothyroidism was made. Postoperative pathology confirmed well-differentiated ectopic thyroid tissue.

**Intervention and outcomes:**

The patient received preoperative levothyroxine (25 μg/day). Under general anesthesia, with laryngoscope support, a partial resection was performed using an electrohook, which enabled simultaneous cutting and hemostasis. The procedure lasted 45 min and was associated with minimal blood loss (20 mL). Postoperatively, she received antibiotics and continued thyroid hormone replacement therapy. Her symptoms resolved completely, and recovery was uneventful. At the 3-month follow-up, she remained asymptomatic, with normalized thyroid function (TSH 5.2 μIU/mL) and an improved quality of life.

**Conclusion:**

Electrohook resection under laryngoscopic guidance is a simple, cost-effective, and safe treatment for symptomatic lingual ectopic thyroid, particularly suitable for primary hospitals, and may improve treatment accessibility for this rare condition.

## 1 Introduction

Ectopic thyroid tissue is a congenital abnormality resulting from failure of normal thyroid descent, with an estimated incidence of 1 in 100,000–1/300,000 in the population and a female predominance (3–8:1) (1). Among all cases of congenital ectopic thyroid tissue, the lingual type accounts for up to 90%, with lesions typically located near the foramen cecum at the base of the tongue. Although the underlying etiology for this congenital anomaly remains unclear, it is believed that mutations in transcription factors involved in thyroid germ cell migration play a key role (2). Most patients with ectopic thyroid are clinically asymptomatic; however, progressive enlargement of the ectopic gland may produce symptoms such as a pharyngeal foreign body sensation, dysphagia, airway obstruction, and dysphonia, with hypothyroidism present in up to 30% of cases (3).

Asymptomatic patients are initially treated with levothyroxine therapy. However, this approach is considered ineffective in patients with large, symptomatic lesions (4). Surgical intervention is indicated in cases of significant airway obstruction or dysphagia. Transoral robotic surgery offers advantages for base-of-tongue masses, including three-dimensional high-definition visualization and enhanced instrument flexibility (5), while coblation destroys tissues using radiofrequency energy and works well at lower temperatures (6). Transoral laser microsurgery (TLM) with a CO₂ laser has been used for lingual thyroid excision, offering precise tissue cutting and simultaneous hemostasis (7).Despite their benefits, these advanced techniques are limited by high costs: robotic systems typically cost 2–3 million US dollars, CO₂ laser systems range from 30,000 to 200,000 US dollars and require additional laser safety infrastructure, and coblation tools cost 70,000–110,000 US dollars.

The technique involves the use of high-frequency electrical current delivered by an electrocautery hook, a long-established instrument in electrosurgery used across general surgery, gynecology, and urology, to achieve tissue dissection tissues and hemostasis (8). This report describes the successful resection of lingual ectopic thyroid using an electrocautery hook under laryngoscopic visualization, demonstrating the feasibility and cost-effectiveness of this technique.

## 2 Case report

A 56-year-old woman presented to the otolaryngology outpatient department in September 2024 with a 10-year history of pharyngeal foreign body sensation and dysphagia, which had progressively worsened over the preceding 3 months. The patient initially developed mild pharyngeal irritation in 2014. No medical consultation was sought during the subsequent 10 years, and the patient self-managed symptoms with over-the-counter throat lozenges, without significant improvement. However, over the preceding 3 months, dysphagia for solid foods had significantly worsened, with symptoms most prominent in the supine position, substantially impairing sleep quality and daily activities. She denied dyspnea, dysphonia, hemoptysis, or odynophagia. No significant past medical history was noted. She was postmenopausal (gravida 2, para 2) and had no significant family history of thyroid illness. A detailed clinical timeline for the patient is provided in Table 1.

**Table 1:** Timeline of clinical course.

Date/time point	Clinical event
2014	Symptom onset: mild throat discomfort and foreign body sensation
2014–2024	Progressive worsening of symptoms; no medical consultation was sought; self-managed with over-the-counter throat lozenges without significant improvement
July 2024	Marked exacerbation: severe dysphagia for solid foods, symptoms worse in the supine position
September 2024	Hospital admission with preliminary diagnosis of a mass at the tongue base
9–22 September2024	Comprehensive diagnostic workup and preoperative preparation: laboratory testing, imaging studies, levothyroxine (25 μg/day) therapy
23 September 2024	Electrohook resection under suspension laryngoscopy (45 min, 20 mL of blood loss)
24 September 2024 (POD 1)	Mild throat pain (VAS score 3/10), marked improvement of foreign body sensation
27 September 2024 (POD 4)	Laryngoscopic examination showing a healing wound with a white pseudomembrane
29 September 2024 (POD 6)	Hospital discharge with complete resolution of symptoms
Postoperative period	
2 weeks postoperative	Telephonic follow-up: complete resolution of symptoms, no discomfort
1 month postoperative	Telephonic follow-up: TSH 5.2 μIU/mL, normalized FT3/T4, significantly improved quality of life
3 months postoperative	Telephonic follow-up: sustained improvement, stable thyroid function

**Figure 1:**
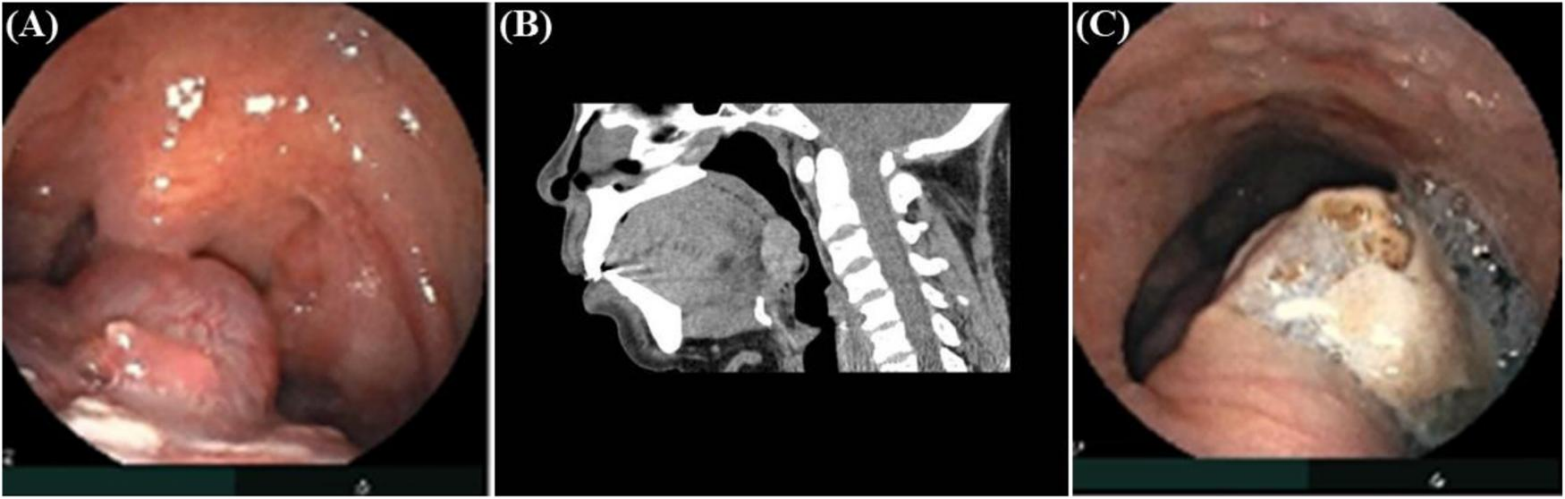
Preoperative and postoperative findings. **(A)** Preoperative laryngoscopy showing a pale red, smooth-surfaced mass at the tongue base. **(B)** Sagittal CT scan demonstrating a hyperdense lesion at the tongue base with well-defined margins. **(C)** Postoperative laryngoscopy on day 4 showing the surgical wound covered with a white pseudomembrane.

Vital signs were stable. Palpation of the neck showed no mass in the normal thyroid position, the trachea was midline, and there were no enlarged lymph nodes. Electronic laryngoscopy showed a pale red, smoothly surfaced, rounded mass at the center of the tongue base, measuring approximately 1.7 cm by 1.6 cm, with a base width of about 1.2 cm. The overlying mucosa was intact, with no evidence of ulceration or hemorrhage (Figure 1A). Sagittal CT imaging further demonstrated the location and morphology of the lesion (Figure 1B).

Laboratory investigations revealed abnormal thyroid function: thyroid-stimulating hormone (TSH) was elevated at 40 μIU/mL (normal range 0.27–4.20 μIU/mL), total thyroxine was 17.75 ng/mL (normal range 65–155 ng/mL), and free triiodothyronine was 3.5 pmol/L (normal range 3.10–6.80 pmol/L). These results are consistent with a diagnosis of hypothyroidism. Plain CT scanning of the oropharynx revealed a nodular hyperdense lesion at the base of the tongue, measuring 2 cm in length, with distinct boundaries and higher density than the surrounding tissues. Neck ultrasonography revealed that the thyroid was markedly reduced in its normal cervical position, with heterogeneous echogenicity.

**Figure 2:**
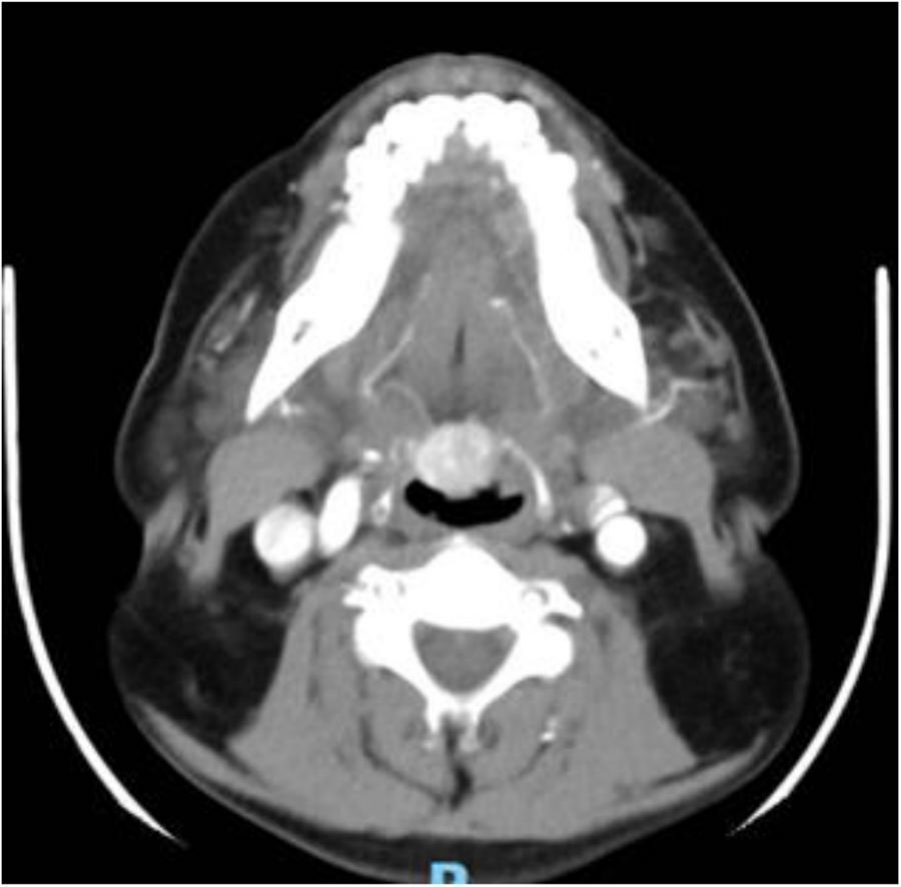
Contrast-enhanced computed tomography findings. Axial view showing an oval hyperdense lesion (1.6 × 1.7 cm) with marked arterial phase enhancement (approximately 85 Hounsfield units) and decreased venous phase enhancement (approximately 45 Hounsfield units), consistent with a characteristic thyroid tissue perfusion pattern.

Further evaluation with contrast-enhanced CT of the oropharynx showed an oval, hyperdense mass in the posterior midportion of the tongue base, with maximal dimensions of 1.6 cm × 1.7 cm. On arterial-phase contrast-enhanced scanning, the lesion showed marked contrast (approximately 85 Hounsfield units), with reduced concentrations during the venous phase (approximately 45 Hounsfield units), consistent with the characteristic “rapid inflow, slow outflow” pattern of the thyroid gland (Figure 2). Thyroid scanning with radiocolloids was not available at our institution. Given the deep location and prominent vascularity of the lesion, fine-needle aspiration biopsy carried a significant risk of hemorrhage. Combined with the patient's refusal to undergo the procedure, fine-needle aspiration cytology wasnot performed.

Based on clinical presentation, high TSH levels (40 μIU/mL), lack of thyroid tissue in the neck, and the typical post-CT enhancement pattern, a primary diagnosis of lingual ectopic thyroid presenting as hypothyroidism was made. Tongue base tumors and lingual cysts were excluded based on the post-CT enhancement pattern.

Given the lesion size of 1.7 cm, severe presentation, and lack of response to conservative management for 10 years, surgical intervention was warranted. Informed consent was obtained from the patient. During the preoperative preparation phase, the patient was given oral levothyroxine (25 μg/day) for 2 weeks to improve thyroid function. The patient fasted for 8 h and was classified as ASA Class II.

**Figure 3:**
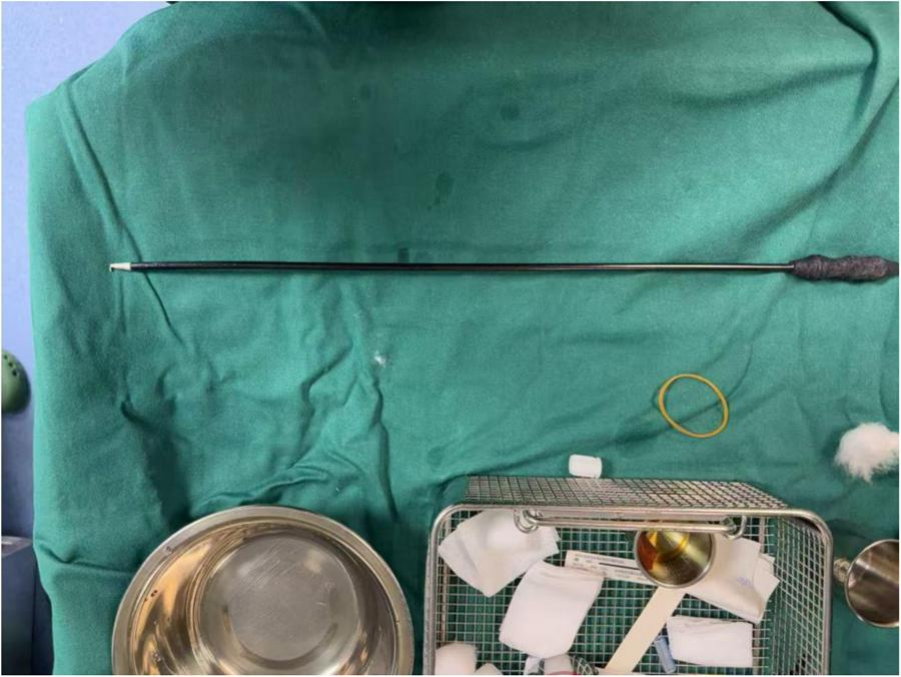
Monopolar electrocautery hook used in the procedure.

On 23 September 2024, the patient underwent partial excision of the tongue base mass using an electrocautery hook under laryngoscopic guidance. The procedure was performed under general anesthesia with oral endotracheal intubation. The patient was positioned supine with the neck extended at approximately 30–40°s. After induction of general anesthesia, a Lindholm-type suspension laryngoscope was inserted and secured to a support frame, allowing full exposure of the tongue base mass in the midline of the field. On direct visualization, the tongue base mass was centrally located at the tongue base, measuring approximately 1.7 cm × 1.6 cm × 1.5 cm. It appeared pale red with a smooth surface, had a base width of about 1.2 cm, and was highly vascular. The procedure was performed using a monopolar electrocautery hook (Figure 3), with current settings of 35 W for cutting and 25 W for coagulation in blended mode. The resection line was marked approximately 2–3 mm from the edge of the tumor, and the plan was to remove the portion that protruded from the tongue base surface while preserving as much deep ectopic thyroid tissue as possible to minimize postoperative hypothyroidism. Starting from one edge of the tumor base, the electrocautery hook was used to incise the mucosa and superficial tissue circumferentially around the lesion. Electrocautery was used to immediately coagulate bleeding points. The entire lesion was separated from the muscle layer of the tongue, with immediate coagulation of all bleeding points by electrocautery and preservation of the lingual artery. The completely resected lesion measured approximately 1.5 cm × 1.3 cm × 0.8 cm and was immediately placed in 10% formalin solution and sent for pathological examination. Examination of the surface revealed that multiple bleeding points ,likely due to previous intubation-relate injury; all bleeding points were appropriately electrocautered. The surface of the lesion was found to be concave, measuring approximately 1.2 cm × 1.0 cm, and was left open to heal by secondary intention. The surgery lasted 45 min, with a blood loss of about 20 mL. Not blood transfusion was required, and there were no intraoperative complications. Postoperatively, the patient was observed in the recovery room for 2 h. After complete awakening and adequate restoration of spontaneous breathing, the endotracheal tube was successfully removed, and the patient was returned to the ward.

**Figure 4:**
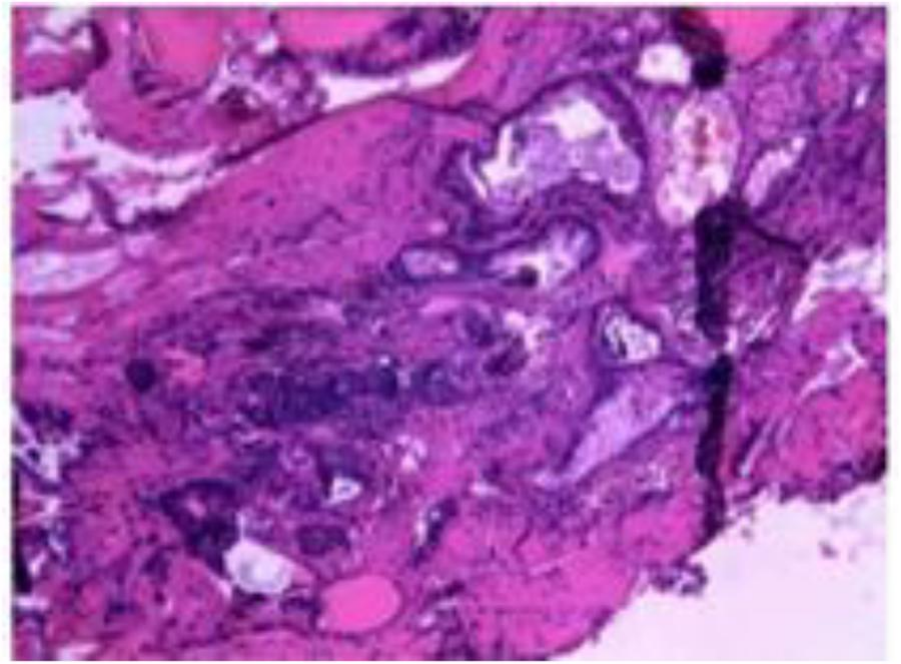
Histopathological examination (hematoxylin and eosin staining, ×200 magnification). Microscopic features showing well-differentiated thyroid follicles of varying sizes filled with eosinophilic colloid. Follicular epithelial cells are cuboidal to columnar, with round nuclei, no cellular atypia, and rare mitotic figures, confirming the presence of ectopic thyroid tissue.

Gross pathological findings revealed gray-red tissue measuring 1.5 cm × 1.3 cm × 0.8 cm. Histopathological examination with H&E staining revealed well-differentiated thyroid follicles of varying sizes, with lumina containing pink-staining material resembling colloid. The follicular epithelial cells were unremarkable and lacked mitotic activity. The pathological findings were consistent with ectopic thyroid tissue (Figure 4).

Postoperatively, feeding was gradually advanced from liquid to normal diet. Medical management included intravenous cefuroxime for infection prophylaxis (twice daily for 3 consecutive days), an increase in oral levothyroxine from 25 to 50 μg/day, as partial resection of the ectopic thyroid tissue was expected to further reduce the total functional thyroid volume in a patient already presenting with significant hypothyroidism (preoperative TSH 40 μIU/mL) and a markedly atrophic cervical thyroid gland, budesonide nebulization, and chlorhexidine mouthwashes. By postoperative day 1, the patient had stable vital signs; she reported mild sore throat [visual analog scale (VAS) 3/10], but the sensation of a foreign body in the pharynx had significantly reduced compared with preoperative levels. She was able to ingest lukewarm liquids, with no evidence of bleeding and shortness of breath. Over time, the patient gradually became asymptomatic. By postoperative day 4, follow-up electronic laryngoscopy revealed a surgical site of approximately 1.0 cm × 0.8 cm, covered with a white pseudomembrane (fibrinous exudates), without evidence of bleeding or purulent drainage; mild hyperemia of the surrounding mucosa was noted (Figure 1C). By postoperative day 6 (29 September 2024), the patient had fully recovered, with complete resolution of the foreign body sensation in the anterior throat restoration of normal swallowing mechanism, and was therefore discharged from the hospital.

The patient was discharged on levothyroxine 50 μg/day, with advice for periodic thyroid function monitoring. At the 2-week telephonic follow-up, the patient reported complete resolution of all her symptoms without any discomfort. She remained asymptomatic at the 1-month telephonic follow-up. Thyroid function tests conducted at a local hospital revealed a serum thyroid-stimulating hormone of 5.2 μIU/mL, with normal free triiodothyronine and thyroxine levels. At the 3-month telephonic follow-up, the patient continued to be asymptomatic with stable thyroid function. However, the patient chose not to continue further telephonic follow-up due to distance constraints. She expressed satisfaction in a statement, describing: “For the first time in 10 years, my throat feels so comfortable. The surgical trauma was minimal, and the recovery was very quick. I am very grateful to the doctor; my quality of life has improved considerably.”

A comparison of the cost and availability of the electrocautery hook surgical method employed in this case with other major surgical methods is presented in Table 2.

**Table 2:** Comparison of surgical techniques for lingual ectopic thyroid resection.

Technique	Equipment cost (US dollars)	Consumable cost (US dollars/case)	Technical requirements	Primary hospital feasibility
Traditional open surgery	Minimal (<$500)	<$100	Standard surgical training	High
Transoral robotic surgery (TORS)	$2,000,000–3,000,000	$1,400–2,800	Specialized robotic surgery training and certification	Very low (tertiary centers only)
Coblation ablation	$70,000–110,000	$280–420	Specialized training in coblation technology	Low (well-resourced hospitals only)
Transoral laser microsurgery (TLM)	$30,000–200,000	$50–150 (laser fiber/consumables)	CO_2_ laser training and laser safety certification	Low (requires laser equipment and safety infrastructure)
Electrohook resection	<$150	$0–28 (reusable or disposable)	Standard electrosurgery training	Very high (universal availability)

## 3 Discussion

This case report presents an effective approach to the excision of lingual ectopic thyroid using an electrocautery hook, particularly relevant in resource-limited healthcare settings. The clinical presentation in this case was consistent with previously reported cases, where chronic symptoms are often associated with hypothyroidism (9). Regarding diagnosis, thyroid radionuclide scanning is considered the gold standard (10), while single-photon emission computed tomography combined with CT scanning can provide more precise localization (11). In this case, the diagnosis was established based on the characteristic enhancement pattern observed on contrast-enhanced CT scanning, supported by postoperative pathological examination (12). The persistence of symptoms despite 10 years of conservative treatment indicated the need for surgical excision.

The evolution of surgical approaches reflects advances in minimally invasive surgical techniques, while also highlighting the reality of the unequal distribution of medical resources. Conventional surgical care has been increasingly supplanted by minimally invasive methods due to the increased surgical trauma associated with traditional approaches (13). Transoral robotic surgery, with its three-dimensional high-definition visualization and flexible robotic arms, offers precision and minimally invasiveness in the surgical treatment of deep structures, such as the tongue base (14). This technology has shown good therapeutic outcomes in lingual ectopic thyroid excision (15) and can be safely performed even in larger or more complex cases (16). However, despite their high efficacy, the high costs of these technologies remain a major obstacle to their use. TORS costs approximately 2–3 million US dollars, with an additional per-case expense of 1,400–2,800 US dollars per case, and coblation costs around 70,000–110,000 US dollars, with a per-procedure cost of 280–420 US dollars. Such disparities in technology costs and the availability of financial resources mean that many patients fail to receive adequate care on time. TLM using CO_2_ laser is another minimally invasive option for tongue base surgery. Howard et al. reported the Mayo Clinic experience with nine lingual thyroidectomies, of which four were performed by TLM and three using TORS; all patients reported significant symptomatic improvement (7). The mean operative time was 91 ± 16 min for TLM, compared with 109 ± 35 min for TORS. However, TLM also has notable limitations, particularly in resource-limited settings. CO_2_ laser systems for surgical use typically cost between 30,000 and 200,000 US dollars and require additional safety measures, including laser-specific protective equipment, a dedicated laser nurse, and specialized training in laser surgery (17). Furthermore, the straight-line delivery of the CO_2_ laser beam through an operating microscope offers limited flexibility when manipulating tissues in the deep tongue base, compared with the articulated robotic arms of TORS systems. While TLM is less expensive than TORS, its equipment costs and safety infrastructure requirements still represent significant barriers for primary hospitals in resource-limited regions.

Direct comparison of postoperative outcomes across surgical techniques for lingual thyroid is limited by the rarity of the condition and the predominance of case reports. In a systematic review by D'Andréa et al. (5), which analyzed 40 reports on lingual thyroid surgery, non-invasive transoral approaches and TORS were associated with significantly fewer tracheostomies comapred with invasive transoral and transcervical approaches (*P* < 0.001); however, surgical complication rates did not differ significantly between techniques. For tongue base surgery in general, postoperative hemorrhage rates have been reported at approximately 1.4% for TLM (18) and 3%–8% for TORS in oropharyngeal procedures (19). Postoperative pain following transoral minimally invasive surgery for tongue base lesions is generally mild, with reported VAS scores of 2–4 in the early postoperative period. In the present case, the patient reported a VAS pain score of 3/10 on postoperative day 1, with only 20 mL of blood loss, suggesting that electrohook resection may achieve comparable short-term outcomes.

In contrast to these advanced but costly techniques, the electrocautery hook, a common monopolar electrosurgical tool, relies on the thermal properties of high-frequency electrical currents, with a mode that allows excision and hemostasis to be completed simultaneously. Monopolar electrosurgery has also been shown to be safe for use in patients with implanted devices such as hypoglossal nerve stimulators (20). The suspension laryngoscope, a basic surgical device used extensively in otolaryngology, has already seen its fair share of large-scale clinical safety and efficiency studies (21). The application of the electrocautery hook in this case demonstrated multiple advantages. The economic advantage is significant, with costs reduced by more than 95% compared with TORS, TLM, and coblation (Table 2). This universal accessibility enables primary healthcare facilities to provide treatment independently, reducing the need for long-distance referrals. Postoperative recovery was smooth, with complete resolution of symptoms and stable thyroid function at the 3-month follow-up, indicating comparable efficacy to advanced techniques with significant cost advantages.

This study has several limitations. As a single case report with only a 3-month follow-up, the level of evidence is low. Radionuclide scanning was not performed. The effectiveness of this approach for larger or deeper lesions remains unclear. Further multicenter studies with longer follow-up are needed to validate its long-term efficacy, safety, and complication rates.

## 4 Conclusion

Electrocautery hook excision successfully treated lingual ectopic thyroid, achieving complete symptom resolution and stable thyroid function at the 3-month follow-up. This technique offers a cost-effective alternative to TORS, TLM, and coblation, with comparable short-term efficacy and universal accessibility. Although multicenter studies are needed to validate long-term outcomes, electrohook excision represents a practical approach for resource-limited facilities and merits further clinical application.

## Data Availability

The raw data supporting the conclusions of this article will be made available by the authors, without undue reservation.
